# Application of a Fluid–Structure Interaction Model for Analysis of the Thermodynamic Process and Performance of Boil-Off Gas Compressors

**DOI:** 10.3390/e21040341

**Published:** 2019-03-28

**Authors:** Bin Zhao, Shuangmei Zhou, Jianmei Feng, Xueyuan Peng, Xiaohan Jia

**Affiliations:** School of Energy and Power Engineering, Xi’an Jiaotong University, Xi’an 710049, China

**Keywords:** BOG compressor, CFD, UDFs, gas pulsation, suction temperature

## Abstract

Boil-off gas (BOG) compressors are among the most critical devices in transportation and receiving systems for liquid natural gas (LNG) because they are used to pump out excess BOG from LNG storage tanks to ensure safety. Because of the ultralow suction temperature, the influence of heat transfer between the cold gas and the compressor parts on the in-cylinder thermodynamic process cannot be ignored. This paper reports the effects of suction temperature on the thermodynamic process and performance of a BOG compressor with consideration of gas pulsation. A computational fluid dynamics (CFD) model with dynamic and sliding meshes was established, in which user-defined functions (UDFs) were used to calculate the real-time valve lift to realize coupling between the thermodynamic process and the gas pulsation, and a performance test rig was constructed to verify the proposed numerical model. The simulated results agreed well with the experimental ones. The results show that as the suction temperature decreased from 30 °C to −150 °C, the first-stage volumetric efficiency decreased to 0.69, and the preheating increased to 45.8 °C. These results should provide academic guidance and an experimental basis for the design and optimization of BOG compressors.

## 1. Introduction

Natural gas (NG) is playing an increasingly important role in the world’s energy structure because of its abundance, high efficiency, high calorific value, and low emission and price. After liquefaction, one cubic meter of LNG generally contains 625 cubic meters of NG in the gaseous state, which makes storage and transportation economical and convenient [1,2,3,4]. Thus, LNG terminals and related projects have developed rapidly in recent years to meet the substantial increase in global demand for NG.

However, owing to heat leakage from the surrounding environment, LNG boils and evaporates into BOG during transportation, storage, and especially unloading of LNG, and the pressure in the container increases as the amount of BOG increases. If the pressure in the container exceeds the designed value, it will pose a severe threat to LNG transportation and the receiving terminals. The excess BOG must be handled to maintain a relatively stable pressure within the container. One conventional method of dealing with excess BOG is to burn it and use its energy to power steam turbines. Another method is to recycle the excess BOG using a BOG reliquefaction plant [5,6].

In general, a compressor must first pressurize the BOG, which is then either directly delivered to pipelines or recycled back into LNG containers after being liquefied in a condenser by mixing it with the subcooled LNG [6,7]. Therefore, the BOG compressor is an essential device for safe handling of BOG, regardless of the technology adopted [8,9].

LNG systems typically use oil-free reciprocating compressors with low suction temperatures between −160 °C and −100 °C as BOG compressors. BOG compressors in LNG terminals are mainly either vertical labyrinth compressors [7] or horizontal piston ring compressors [10]. Also, a multistage compressor with a larger compression ratio is used to achieve a significant pressure difference between the compressor inlet and outlet.

The temperature of BOG in LNG systems can be as low as −160 °C, which leads to a considerable temperature difference between the suction chamber and the surrounding environment. Such a significant temperature difference drives the heat transfer from the surrounding environment to the BOG through the suction pipe or cylinder, resulting in thermal stresses on the cylinder. This heat transfer thus has a significant influence on the in-cylinder thermodynamic process. Therefore, the design of the BOG compressor must consider the effects of low suction temperatures. For example, because the piston comes into direct contact with the cold gas, the piston rings must be made of a self-lubricating material with appropriate friction and wear characteristics.

Many investigators have studied the thermodynamic process and heat transfer in reciprocating compressors. Adair et al. [11] discussed and compared the existing correlations for heat transfer in reciprocating compressors and engines. They performed an experimental investigation of the instantaneous heat transfer rates to the cylinder wall of a reciprocating refrigeration compressor and presented a new correlation that successfully considered the instantaneous heat transfer rates to estimate compressor efficiencies. Chong and Watson [12] presented an accurate procedure for prediction of heat transfer and pressure variations in the cylinder of a low-speed reciprocating air compressor, but they did not consider the influence of residual air motion at the end of the suction stroke and the discharge of the compressed gas. Under various pressure ratios, rotational speeds, and suction temperatures, Liu and Zhou [13] measured the temperature distribution on the cylinder wall of a reciprocating refrigeration compressor and the coefficient of the heat transfer between the cylinder wall and the gaseous refrigerant. Recktenwald et al. [14] used two numerical models to investigate the instantaneous heat transfer between the cylinder walls and gas in a reciprocating compressor. Keribar and Morel [15] presented a new method to predict gas-to-wall heat transfer in reciprocating compressors based on in-cylinder flow velocities. This method can be applied to calculate heat transfer coefficients as a function of speed, pressure ratio, fluid properties, compressor valve, and piston geometry. Fagotti et al. [16] established the most reasonable model after comparing some widely used heat transfer models in a compressor simulation program. Pereira et al. [17] established a simplified two-dimensional finite volume model for numerical analysis of the unsteady in-cylinder heat transfer of a small reciprocating compressor under actual operating conditions, and they concluded that heat transfer was strongly affected by the suction and discharge processes. Ooi [18] analyzed the heat transfer and temperature distribution of a hermetic reciprocating refrigeration compressor using the lumped thermal conductance approach. Jang and Jeong [19] carried out an experimental study on the convective heat transfer between a scroll compressor and a refrigerant. Ooi and Zhou [20] formulated a two-dimensional numerical model to simulate the fluid flow and heat transfer inside the working chamber of a scroll compressor and found significantly higher convective heat transfer between the gas and the scroll wrap walls. Tan and Ooi [21] conducted a theoretical study on convective heat transfer between the working fluid and the surrounding chamber wall for the design of a novel revolving vane (RV) compressor and improved the accuracy of the mathematical prediction of the novel RV compressor. Shen et al. [22] established a three-dimensional finite element model to simulate the cylinder temperature distribution of a BOG compressor. The results demonstrated that the temperature fluctuation on the inner surface of the cylinder was prominent in the first few minutes after startup and that it decreased during steady operation.

In the above literature review, several factors that may affect the heat transfer in compressors were analyzed. The authors explored the suction heating of a BOG compressor using the verified finite element model with dynamic meshes [23]. However, most of these studies did not consider gas pulsation when investigating the heat transfer and thermodynamic process in the compressor. Therefore, this paper presents an investigation that considers gas pulsation, focusing on the thermodynamic process and performance of a BOG compressor. Both simulation and experimental methods were applied to analyze the in-cylinder pressure, temperature variations, and valve movement characteristics of a BOG compressor. Furthermore, the effects of the suction temperature on preheating, the volumetric efficiency, and the valve operating characteristics of the compressor are discussed.

## 2. Numerical Model

Based on the CFD method, a numerical model with dynamic and sliding meshes was established for the internal flow in the first-stage cylinder and its piping system. The thermodynamic process in the cylinder was also correlated with the gas pulsation in the valve chambers via the UFDs obtained from valve dynamics for simultaneous solution of the thermodynamic process, valve dynamics, and gas pulsation. A three-dimensional fluid–structure interaction (FSI) simulation was eventually realized.

### 2.1. Geometric Model

Generally, the suction pressure of a BOG compressor is slightly greater than atmospheric pressure, ~0.01–0.04 MPa, whereas the discharge pressure is about 1.6 MPa. Therefore, at least two stages are required in BOG compression. The first-stage cylinder of a BOG compressor has a lower suction temperature and a relatively larger compression ratio than the second stage, whereas the second-stage suction temperature usually increases to approximately −40 °C after the first-stage compression.

**Figure 1:**
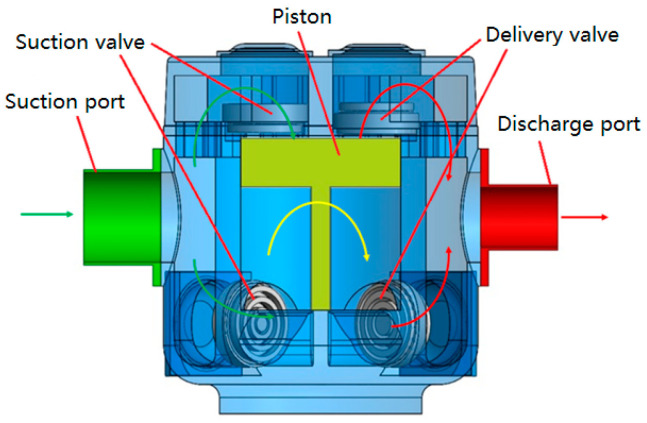
Structure of the first-stage cylinder.

**Figure 2:**
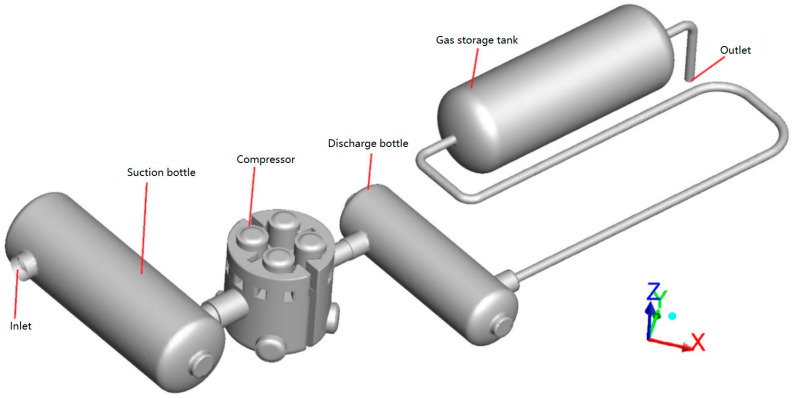
Computational fluid domain consistent with the experimental rig.

The compressor investigated was an L-type two-stage double-acting reciprocating compressor. Taking its first-stage cylinder as the research object, the in-cylinder thermodynamic process, valve movement, and gas pulsation were investigated under low suction temperature conditions. Figure 1 shows the sectional geometry of the first-stage cylinder, including the suction chamber, discharge chamber, piston, piston rod, suction valve, and delivery valve. The computational fluid domain of the first-stage cylinder and the corresponding piping system extracted using ANSYS DM is shown in Figure 2, which is consistent with the experimental rig presented in Section 3.

### 2.2. Fundamental Equations

Three conservation laws govern all heat transfer and fluid flow problems: mass, momentum, and energy conservation laws. The mass conservation equation is

(1) $$\frac{\partial \rho }{\partial t}+\nabla \cdot \left(\rho \vec{u}\right)=0$$

The momentum conservation equation can be expressed in tensor form as follows

(2) $$\frac{\partial \left(\rho \vec{u}\right)}{\partial t}+\nabla \cdot \left(\rho \vec{u}\vec{u}\right)=-\nabla p+\nabla \cdot \left[\mu \left(\nabla \vec{u}+\nabla {\vec{u}}^{T}-\frac{2}{3}\nabla \cdot \vec{u}I\right)\right]+\rho \vec{g}+\vec{F}$$

The energy conservation equation is

(3) $$\frac{\partial \left(\rho T\right)}{\partial t}+\nabla \cdot \left(\rho \vec{u}T\right)=\nabla \cdot \left(\frac{k_{h}}{c_{p}}\nabla T\right)+S_{T}$$

To close the governing equations, the state equation is added. The cubic equation of state, Peng–Robinson equation, and the ideal state equation were both selected to solve the internal flow under low suction temperature. The comparison between the predicted results showed limited discrepancy, while the model with the cubic equation of state consumed more computationally redundant. Therefore, the working fluid was taken as ideal gas, and its equation of state could be written as

(4) $$p=\rho R_{g}T$$

Additional turbulent transportation equations must be considered to perform a numerical simulation. The turbulence model used in this study was a detached eddy simulation (DES) model, which is essentially a hybrid LES/RANS model in which the unsteady Reynolds-averaged Navier–Stokes (RANS) model was used in the boundary layer and the LES model was used in both the core region and the separated region of the turbulent flow.

The transportation equations for the turbulence kinetic energy k and the turbulence dissipation rate ε in the RNG k-ε model are

(5) $$\frac{\partial }{\partial t}\left(\rho k\right)+\frac{\partial }{\partial x_{i}}\left(\rho ku_{i}\right)=\frac{\partial }{\partial x_{j}}\left(\alpha_{k}\mu_{eff}\frac{\partial k}{\partial x_{j}}\right)+G_{k}-\rho \varepsilon$$

(6) $$\frac{\partial }{\partial t}\left(\rho \varepsilon \right)+\frac{\partial }{\partial x_{i}}\left(\rho \varepsilon u_{i}\right)=\frac{\partial }{\partial x_{j}}\left(\alpha_{\varepsilon }\mu_{eff}\frac{\partial \varepsilon }{\partial x_{j}}\right)+C_{1\varepsilon }\frac{\varepsilon }{k}G_{k}-C_{2\varepsilon }\rho \frac{\varepsilon^{2}}{k}$$

In addition, the transportation equations in the DES with the realizable k-ε model are

(7) $$\frac{\partial }{\partial t}\left(\rho k\right)+\frac{\partial }{\partial x_{i}}\left(\rho ku_{i}\right)=\frac{\partial }{\partial x_{j}}\left[\left(\mu +\frac{\mu_{t}}{\sigma_{k}}\right)\frac{\partial k}{\partial x_{j}}\right]+G_{k}-Y_{k}$$

(8) $$\frac{\partial }{\partial t}\left(\rho \varepsilon \right)+\frac{\partial }{\partial x_{i}}\left(\rho \varepsilon u_{i}\right)=\frac{\partial }{\partial x_{j}}\left[\left(\mu +\frac{\mu_{t}}{\sigma_{\varepsilon }}\right)\frac{\partial \varepsilon }{\partial x_{j}}\right]+\rho C_{1}S\varepsilon -\rho C_{2}\frac{\varepsilon^{2}}{k+\sqrt{v\varepsilon }}$$

The turbulent viscosity is computed as

(9) $$\mu_{t}=\rho C_{\mu }\frac{k^{2}}{\varepsilon }$$

where $C_{\mu }$ is constant for the RNG model but not for the DES model. It is calculated by

(10) $$C_{\mu }=\frac{1}{A_{0}+A_{s}\frac{kU^{*}}{\varepsilon }}$$

The governing equations of LES are obtained by filtering the transient Navier–Stokes equations through a filter function, effectively filtering out the eddies whose scales are smaller than the filter width or grid size. The filter process implicit in the finite volume method (FVM) itself is

(11) $$\bar{\phi }\left(x\right)=\frac{1}{V}\int_{v}\phi \left(x^{\prime }\right)dx^{\prime },x^{\prime }\in v$$

The filter function implied in Equation (11) is

(12) $$G\left(x,x^{\prime }\right)=\{\begin{array}{ll} 1/V, & x^{\prime }\in v\\ 0, & x^{\prime }\notin v \end{array}$$

The compressor valves that are located between the working and valve chambers are all automatic. To achieve a coupling simulation of the thermodynamic process and gas pulsation, the corresponding valve dynamics equations must be established to connect the pressures in the working and valve chambers. In this paper, the collisional stresses of the valve plates are ignored.

During the operation of a compressor, the valve plate periodically reciprocates between the seat and lift guard under the combined action of gas and spring forces. As the gas flows through the valve channels and collides with the valve plate it is forced to change direction and flow out of the gap between the valve plate and seat, which is equivalent to being throttled and losing energy. Therefore, the valve design directly affects the compressor’s economy. Without considering the inclination of the valve plate, the valve plate in an oil-free compressor can be regarded as a single degree-of-freedom mass–spring system. The dynamic model of a valve plate is given by

(13) $$ma=F_{p}-F_{o}$$

where m is the lumped mass obtained by the valve plate mass $m_{v}$ plus one-third of the spring mass $m_{s}$.

The summation of the gas forces acting on a valve plate, $F_{p}$, can be calculated by

(14) $$F_{p}=\int_{A}pdA$$

where p is the gas pressure acting on the surface of the valve plate.

The dynamic spring force $F_{s}$ during the movement of the valve plate can be calculated as

(15) $$F_{s}=NK\left(H_{s}+H\right)$$

where N is the number of springs corresponding to a valve plate, $H_{s}$ is the precompression of each spring, and H is the valve lift.

### 2.3. Computational Domain

**Figure 3:**
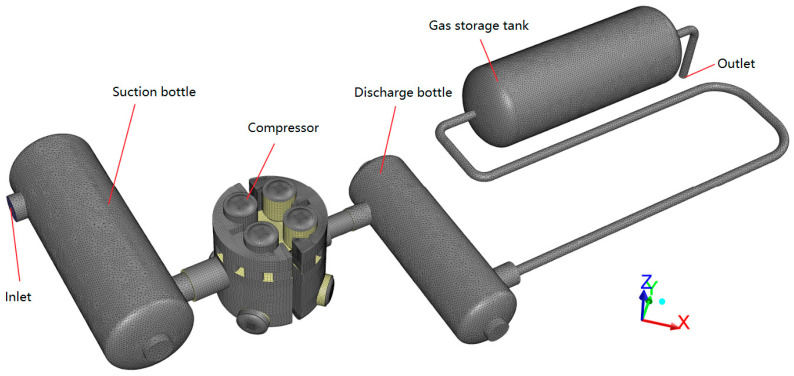
Computational fluid dynamics (CFD) model of the internal flow in a boil-off gas (BOG) compressor and its piping system.

The internal flow in the compressor was discretized using structured grids. The internal flows in the remaining devices were discretized using unstructured grids near their walls and hexahedral structured grids at their cores. The CFD model using the FVM is shown in Figure 3. There were 2.21 × 10^6^ elements in the discrete model, in which the number of compressor elements was 1.76 × 10^6^ and the number of all other elements was 0.45 × 10^6^. A mesh independence study had already been conducted.

### 2.4. Boundary and Initial Conditions

Temperature boundary conditions of −150 °C and −30 °C were used at the inlet of the suction bottle and the outlet of the gas storage tank, respectively, and the corresponding pressure boundary conditions were 0.02 MPa and 0.2 MPa. Moreover, the lumped parameter model was used to determine the initial opening time of the suction and delivery valves.

Because the piston in the discrete model was at the top dead center (TDC), the initial in-chamber pressure and temperature of the head side were set as the discharge pressure and temperature, and those of the shift side were set as the suction pressure and temperature. The UDFs were used to set the piston speed corresponding to the crank angle, and they updated the valve lift in real time.

### 2.5. Solution Method

The PISO algorithm, which can enhance the computational efficiency, was applied to solve the governing equations in the simulation. Moreover, the Green–Gauss method was adopted to interpolate the gradient of the diffusion term. The PRESTO scheme was adopted for the pressure interpolation. A second-order upwind scheme was adopted for the convection term, the turbulent kinetic energy, and the turbulent dissipation rate. Based on the rotational speed of 420 rpm, a time step of 9.92 × 10^−5^ s corresponding to a crank angle interval of 0.25° was considered sufficient to evaluate the thermodynamic process, gas pulsation, and valve motion.

Using 24 cores for parallel solving, each cycle took about 21 h, and five or six cycles were needed to make the calculation converge. During the first three cycles of the solution process, the valve opening and closing times required updating.

## 3. Experimental Investigation

### 3.1. Experimental Facilities

A test rig was built by taking an oil-free reciprocating compressor, whose structure is shown in Figure 1, to study the BOG compressor performance under low suction temperatures. The technical parameters of the compressor are listed in Table 1. Nitrogen was selected as the working fluid because of the flammability and explosive nature of natural gas. 

**Figure 4:**
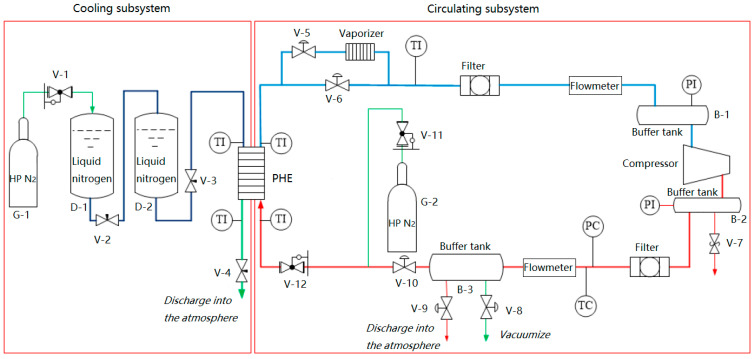
Schematic diagram of the test rig.

A schematic diagram of the test rig is shown in Figure 4. The test rig consisted of two parts: cooling and circulating. After being decompressed by pressure relief valve V-1, high-pressure (HP) nitrogen gas from gas bottle G-1 was injected into the liquid nitrogen storage tank to power the liquid nitrogen flow in the cooling subsystem. The nitrogen gas discharged from the compressor in the circulating subsystem was cooled in the plate heat exchanger (PHE) with the latent heat of liquid nitrogen vaporization and partial sensible heat after evaporation.

The pressurizer of the liquid nitrogen storage tank cannot produce a stable liquid feeding pressure in actual use, so the pressure in Dewar D-1 was adjusted by pressure relief valve V-1 to control the liquid nitrogen flowrate through the PHE, thereby realizing rough control of the compressor suction temperature. Accurate adjustment of the suction temperature was further achieved when the cooled nitrogen gas flowed through the vaporizer. Buffer tanks B-1 and B-2 were used to reduce the effects of gas pulsation.

**Figure 5:**
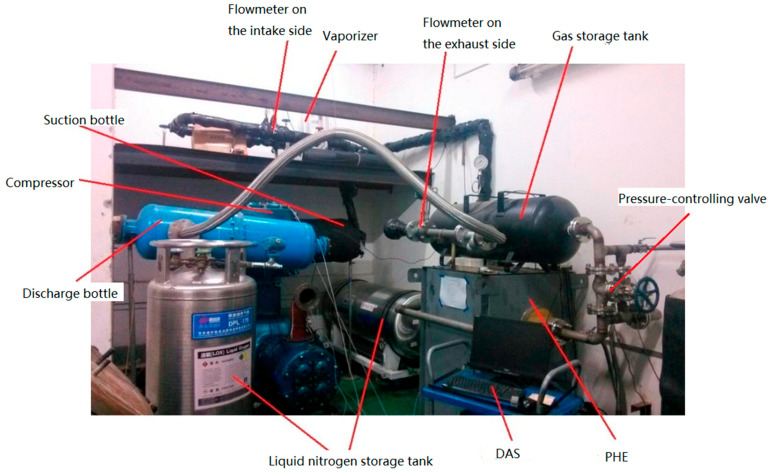
The built test rig.

The test rig was built as shown in Figure 5. After flowing through the PHE, the nitrogen can be cooled to as low as −178 °C, so it was necessary to insulate the pipes and suction bottle (i.e., buffer tank B-1) from the PHE to the compressor suction port. The piping from the compressor discharge port to the PHE required no insulation because of the higher discharge temperature.

After being vacuumed from multiple points and then filled with nitrogen gas, the circulating subsystem was precooled to reduce the time required for reaching steady operation and decrease thermal stresses on components such as cylinders. It should be noted that before startup, the pressure in the circulating subsystem should be slightly higher than the atmospheric pressure. After startup, with the gradual increase of the compressor discharge pressure, it was necessary to continue adding nitrogen gas into the circulating subsystem while adjusting the pressure control valve until the desired operation was achieved. As the compressor suction temperature gradually decreased, the average gas density increased and the compression ratio decreased, which made it necessary to refill the subsystem with nitrogen gas to continue the desired operation.

### 3.2. Experimental Setup

An important parameter to verify the accuracy of the numerical model is pressure, such as dynamic pressures in the suction and discharge chambers and the working chamber. CTL-190M-3.5BarA dynamic pressure sensors, whose main technical parameters are shown in Table 2, were selected to detect these pressures. This kind of pressure sensor is an absolute pressure sensor that is especially suitable for gas dynamic pressure measurements at ultralow temperatures. It can still be used when the pressure to be tested exceeds its rated range. A pressure sensor mounted at the center of the cylinder head was used to measure the dynamic pressure in the head-side working chamber. The dynamic pressures in the suction and discharge chambers can reflect the gas pulsation characteristics in the valve channels.

**Figure 6:**
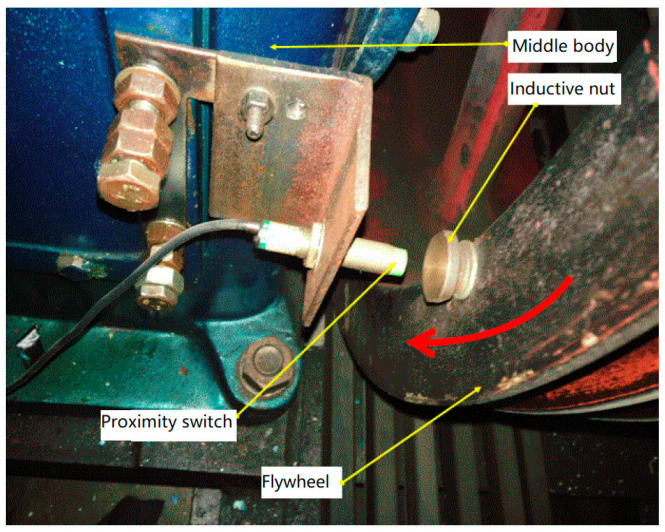
Arrangement of inductive nut and proximity switch.

The piston position should be determined in real time to draw the *p–V* diagram, which is the relationship between the in-cylinder dynamic pressure and the working volume. Therefore, the flywheel connected to the crankshaft was rotated to a position at which the piston moved to the TDC. Then, an inductive nut was mounted on the flywheel as a marker of the TDC signal, and a proximity switch installed near this position was regarded as a TDC sensor (see Figure 6). When the flywheel was at this position, the TDC sensor would output a pulse signal indicating that the piston moved to the TDC. In this way, it was easy to clarify the correspondence between the piston position (or crank angle) and the dynamic pressure. Under the condition that a pulse signal can be generated, the distance between the switch and the nut was increased as much as possible to avoid possible collisions between the two during operation.

**Figure 7:**
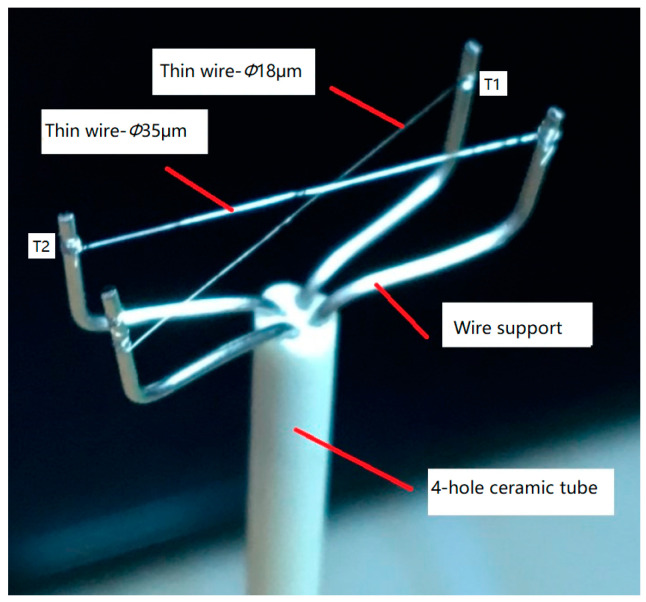
Structure of the two-thermocouple probe used in the experiment [24].

It is known from the gas state equation that two state parameters are required to describe the state of the working fluid in a compressor. One is the pressure, which was conveniently measured as described above. The other is the fluid temperature, which is easier to measure than the remaining state parameters such as density and enthalpy. A two-thermocouple probe, shown in Figure 7, was used to detect the transient fluid temperatures before and after the suction valve.

Moreover, several T-type thermocouples were installed to monitor steady-state temperatures, mainly that of the gas before the compressor inlet flange, that at the inner wall of the suction chamber, that at the inner wall of the discharge chamber, and that of the gas after the compressor outlet flange.

The valve lift was measured using a high-precision eddy current sensor KD2306-9U with a response speed of up to 1 MHz. It can work under low-temperature conditions, which met the experimental requirements. Its measurement range is 0 to 4 mm, corresponding to the output voltage range of 0 to 10 V. For easy access to the wires, the eddy current sensors were mounted on the seat of the suction valve and the lift guard of the delivery valve. It was necessary to calibrate the eddy current sensor before measurement because its output voltage is related to the material of the object being tested. Because the output is linear with the displacement of the part being tested, the valve lifts at the seat and lift guard can be used for calibration.

An integrated target flowmeter JGLBS-50-32-242 with temperature–pressure compensation was selected to measure the flowrate. To ensure an accurate measurement, it is generally necessary to install straight pipes before and after the flowmeter, whose lengths are no less than ten times and five times the pipe diameter, respectively. The rated range of the flowmeter was 80–800 m^3^·h^−1^, corresponding to an output current range of 4–20 mA.

The power measurements were performed using a NORMA 4000 power analyzer with the ability to analyze DC and several megahertz AC. The maximum voltage and current that the power analyzer can measure are 1000 V and 20 A, respectively, so the current and voltage curves can be accurately tracked to calculate the corresponding power with a maximum error of 0.3%. Because the current to be measured was much higher than 20 A, a clamp-on ammeter was added to expand the measuring range of the current.

**Figure 8:**
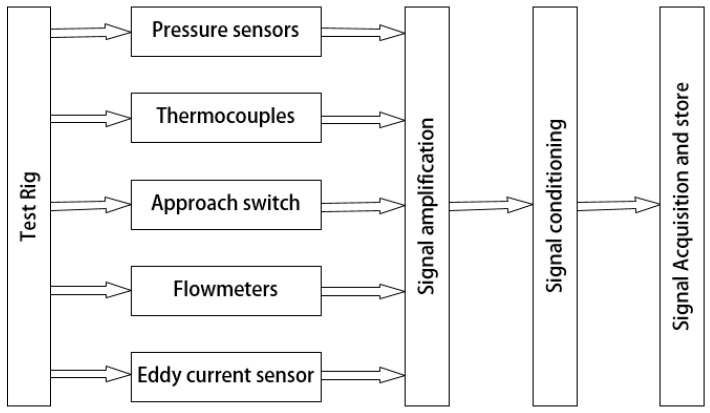
Flow chart of the data acquisition system (DAS) in the experiment.

In a data acquisition system (DAS), the analog signals output by sensors, such as current or voltage, are converted into digital signals for storage in a computer. A flowchart of the DAS adopted in this experiment is shown in Figure 8. The maximum rated output level of the TDC sensor (~5 V) was far higher than that of the pressure sensor (100 mV). Therefore, to avoid signal interference, the output of the TDC sensor was attenuated by a resistor divider before being input to the DAS to match the maximum rated output of the pressure sensor.

The DAS used in this study mainly included a signal-conditioning device—NI SCXI-1125—and an acquisition card—PCI-6220—with 16 analog inputs and a 250 kHz sampling frequency.

## 4. Results and Discussions

### 4.1. Comparison of Simulation and Experimental Results

The following comparisons were made to verify the accuracy of the FSI model at a suction temperature of −150 °C.

#### 4.1.1. *P–θ* Diagrams in the Cylinder

**Figure 9:**
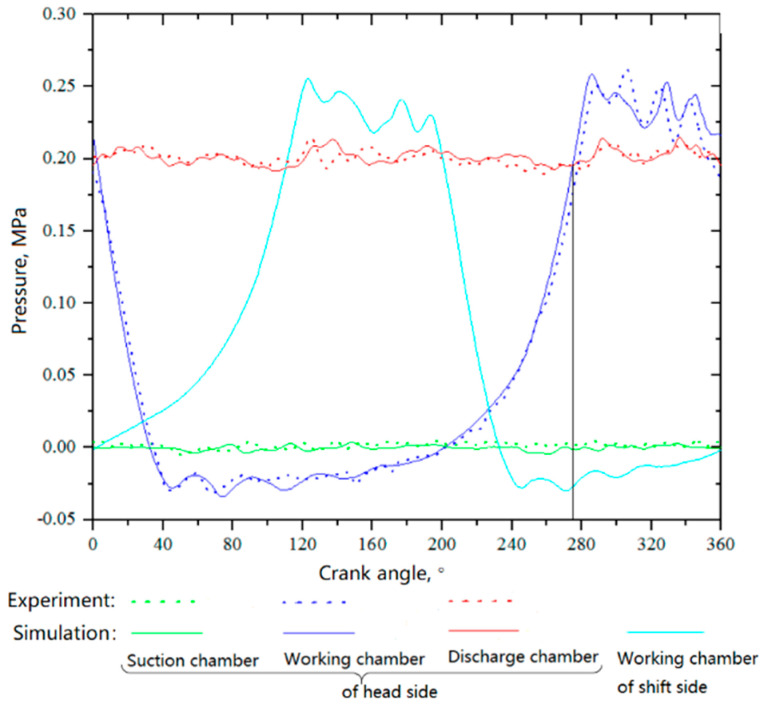
*p–θ* diagrams in the cylinder (suction temperature: −150 °C).

Figure 9 shows the pressure curves in the suction, working, and discharge chambers at a suction temperature of −150 °C. As can be seen from the figure, the simulation results agreed well with the experimental results, except that the pressures in the working chamber were somewhat different during the discharge process. 

Because the buffer tanks, which were located before and after the compressor, had the effect of reducing the gas pulsation, the nonuniformities of the suction pressure obtained by simulation and experiment were 7.9% and 10.02%, respectively, and the nonuniformities of the discharge pressure obtained by simulation and experiment were 8.78% and 8.33%, respectively.

**Figure 10:**
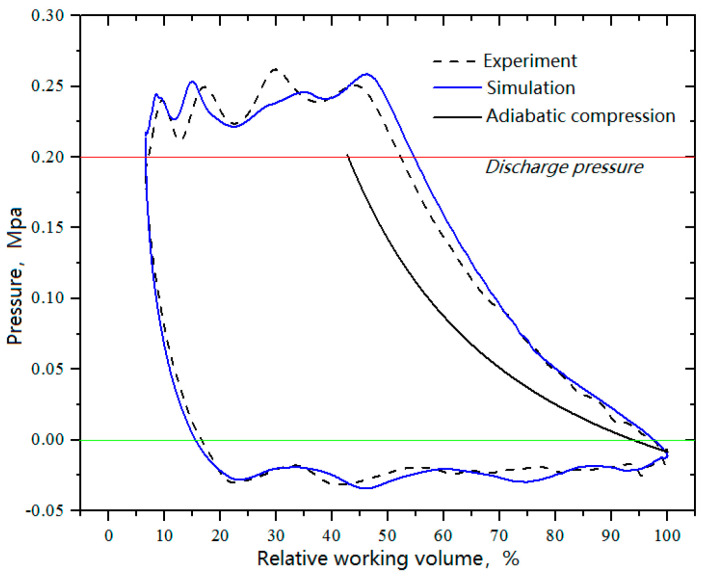
*p–V* diagrams in the working chamber of head side (suction temperature: −150 °C).

Figure 10 shows the *p–V* diagrams in the working chamber of the head side according to Figure 9. Thus, the indicated power and power losses can be calculated (see Table 3). The errors between the simulated value and the corresponding experimental value were 4.0% for the indicated power, 6.2% for the power losses of the suction valve, and 7.6% for the power losses of the delivery valve. Moreover, the volumetric efficiency $\eta_{v}$ of the cylinder head side was calculated to be 0.72 by Equation (16) based on the *p–V* diagrams.

(16) $$\eta_{v}=\frac{V_{d}}{V_{s}}\frac{p_{d}}{p_{s}}\frac{T_{s}}{T_{d}}$$

The volumetric efficiency of the first-stage cylinder was 0.69 according to the measured inlet and outlet volume flowrates of 697.9 Nm^3^·h^−1^ and 481.6 Nm^3^·h^−1^. The theoretical adiabatic power $P_{ad}$ was 17.8 kW by Equation (17). The shaft power $P_{sh}$ was 36.2 kW using Equation (18) according to the measured electrical power $P_{e}$. Therefore, the adiabatic efficiency of the first-stage cylinder was ~49.2%.

(17) $$P_{ad}=\frac{q_{v}}{60}\lambda_{\phi }\lambda_{c}p_{s}\frac{\kappa_{T}}{\kappa_{T}-1}\left(\varepsilon^{\frac{\kappa_{T}-1}{\kappa_{T}}}-1\right)\frac{Z_{s}+Z_{d}}{2Z_{s}}$$

(18) $$P_{sh}=P_{e}\cdot \eta_{d}\cdot \eta_{t}\cdot \eta_{m}$$

#### 4.1.2. *T–θ* Diagrams Inside the Cylinder

**Figure 11:**
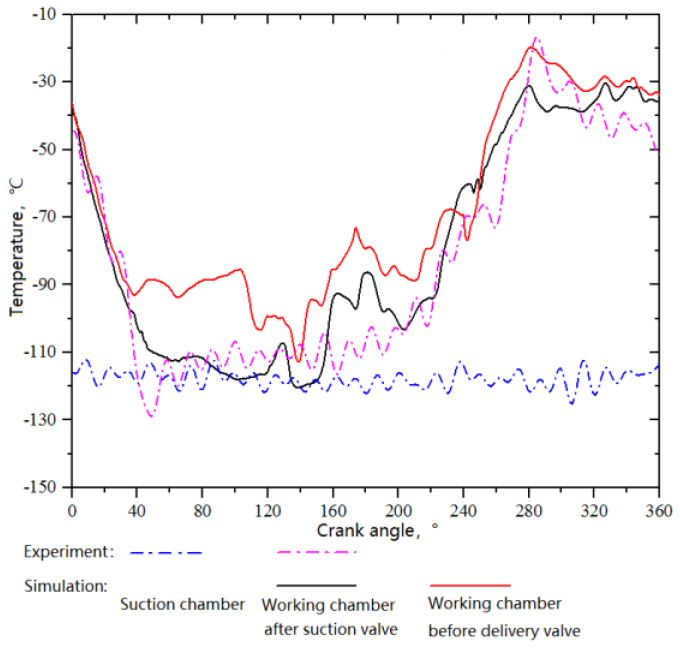
*T–θ* diagrams inside the compressor (suction temperature: −150 °C).

The variations of in-compressor fluid temperatures with crank angle are shown in Figure 11. The in-cylinder fluid temperatures decreased during the expansion, rose slightly during the suction stroke, increased gradually with increasing pressure during the compression, reached a peak, and then decreased slightly during the discharge. With a suction temperature of −150 °C, the fluid temperature before the suction valve fluctuated between −126.1 °C and −112.9 °C, and its average was −121.8 °C. During the suction process, that is, within the crank angle range of 45° to 180°, the in-cylinder fluid temperature after the suction valve varied between −129.6 °C and −103.2 °C, and it was approximately −104.2 °C at the closing of the suction valve. Therefore, the preheating was about 45.8 °C, which is divided into two parts: one was the difference between the compressor suction temperature and the average fluid temperature before the suction valve, ~28.2 °C, and the other was the heating in the valve channels and working chamber, ~17.6 °C. It should be noted that the difference between the experimental and simulated in-cylinder fluid temperature curves around 170° crank angle was mainly induced by the simplified model of the leakage passage. The leakage passage in the piston rings were simplified into an annular channel. Despite the mass flow rate was kept identical, the flow fields of the real model and the simplified model were slightly different, especially in the temperature distribution and history of the monitored point.

**Figure 12:**
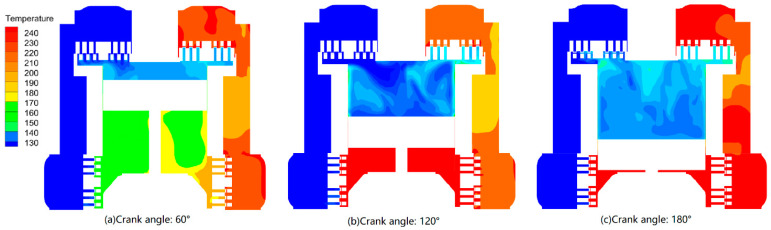
Temperature distributions inside the cylinder (suction temperature: −150 °C).

Figure 12 shows the temperature distributions inside the cylinder. The results indicated that the in-cylinder temperature still increased during the suction process, even without considering heat transfer, because the working chamber of the shift side was compressing when that of the head side was in the suction process. The pressure difference between the two working chambers made the high-temperature gas of the shift head leak to the head side through the clearance between the piston and the cylinder wall, increasing the average gas temperature of the head side.

#### 4.1.3. Lift of Compressor Valve

**Figure 13:**
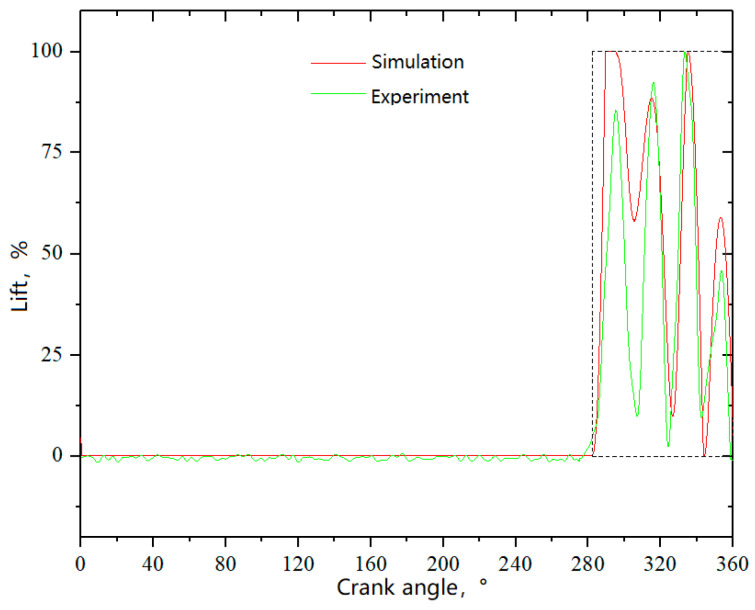
Lift variations of the delivery valve of head side (suction temperature: −150 °C).

In the experiment, only the outer ring lift of the delivery valve, located on the head side, was measured and compared with the simulation results. The lift variations of the outer valve plate under a suction temperature of −150 °C are depicted in Figure 13. Even if the valve movement simulation did not consider the preheating before the suction valve, the consistency between the experimental and simulation results is still high as seen from the valve movement law. However, there was still a more significant deviation in the first pulsation of the valve lift. When the valve plate started to move towards the seat (namely, the initial position) during the first pulsation, it was already very close to its initial position in the experimental results, whereas it returned to the lift guard just after moving a small distance in the simulation. The area enclosed by the dashed line in Figure 13 was the maximum flow area of the valve under ideal conditions. The total flow area obtained by simulation was 57.7% of the ideal value, whereas the experimental result was 41.4%. The simulated and experimental maximum speed of the valve plate were 1.53 m·s^−1^ and 1.72 m·s^−1^, respectively.

**Figure 14:**
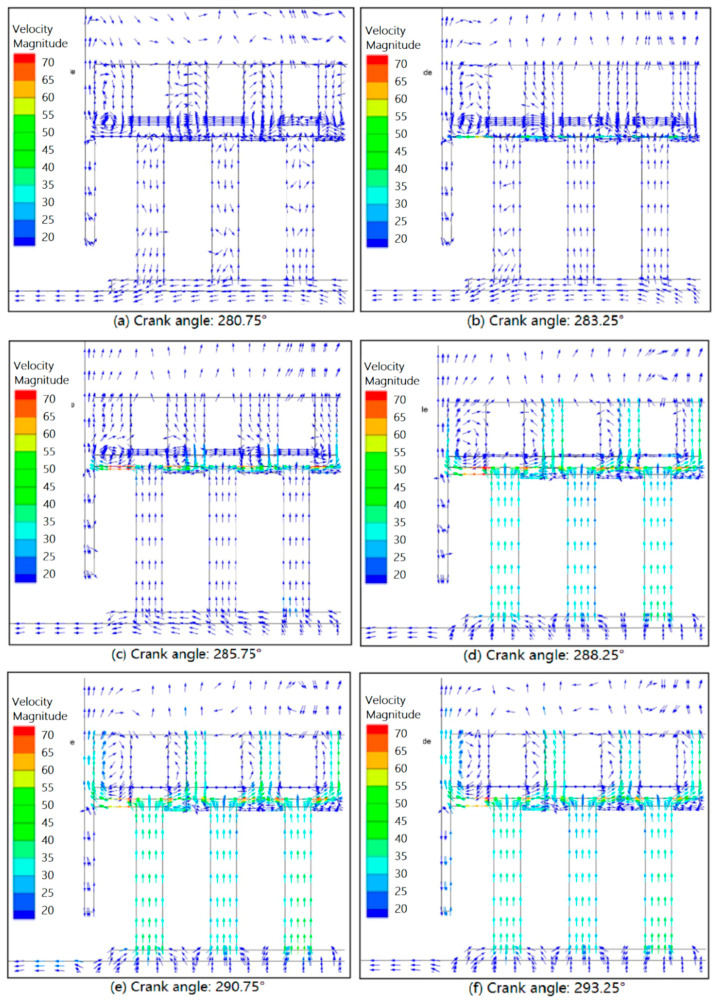
Velocity distributions in the delivery valve of the head side during the opening.

Figure 14 shows the velocity distributions in the delivery valve during the opening. During movement of the valve plate from the seat to the lift guard, the average flow velocity through the valve seat and lift guard gradually increased, and several vortices formed in the valve channels, inducing local resistance loss and reducing the valve flow capacity. The sound speed of the nitrogen was 224.8 m·s^−1^ at a suction temperature of −150 °C. The maximum Mach number in the delivery valve channels was 0.35, corresponding to a maximum flow velocity of 78.5 m·s^−1^.

### 4.2. Characteristics of Steady-State Temperature inside the Compressor

**Figure 15:**
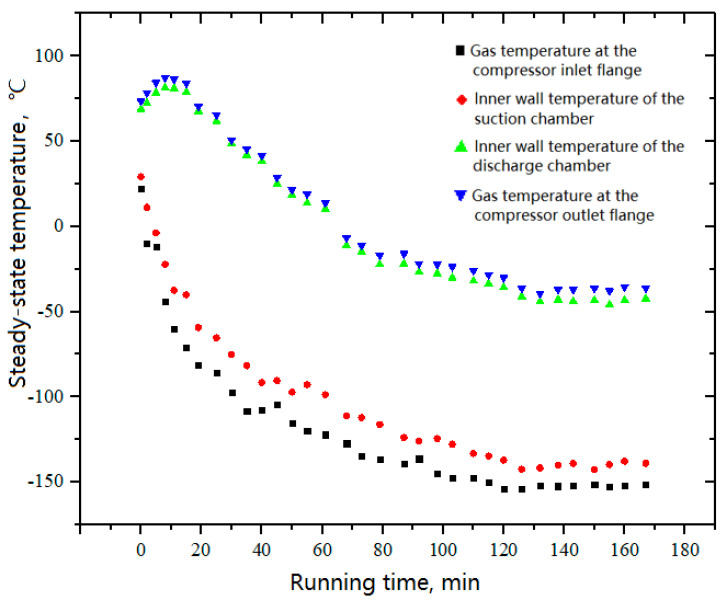
Variations of the in-compressor temperatures with running time.

To achieve a lower suction temperature, liquid nitrogen was used to cool the nitrogen gas in the system. Therefore, the duration of the experiment was limited by the capacity of the liquid nitrogen storage tank. The experiment used three liquid nitrogen storage tanks, including one horizontal tank with a volume of 0.42 m^3^ and two vertical tanks with volumes of 0.175 m^3^. The T-type thermocouples were used to monitor the in-compressor temperatures as a function of the run time. Generally, while the compressor started up at the ambient temperature, the pressure ratio was built gradually with the manually charging of nitrogen. Despite the suction temperature decreased in this process, the discharge temperature would increase with the increase of pressure ratio. It took ~20 min to acquire the expected pressure ratio. Then, the suction temperature continued to decrease and consequently contributed to the decrease of the discharge temperature. Figure 15 shows the variations of the temperature histories inside the compressor during the cooling process. After 120 min, the suction temperature gradually stabilized at −150 °C.

The water vapor in the air condenses and forms frost on the outer surface of the cylinder because the outer wall temperature of the cylinder is much lower than the surrounding dew point temperature. After lengthy operation, a thick layer of frost forms, which can provide excellent insulation. However, when the frost is just forming or the suction temperature is not very low, the frost layer formed is relatively thin with an uneven shape. The initial dendritic growth of the frost layer increases the heat-dissipating surface, acting as fins, that is, improving the heat transfer performance.

**Figure 16:**
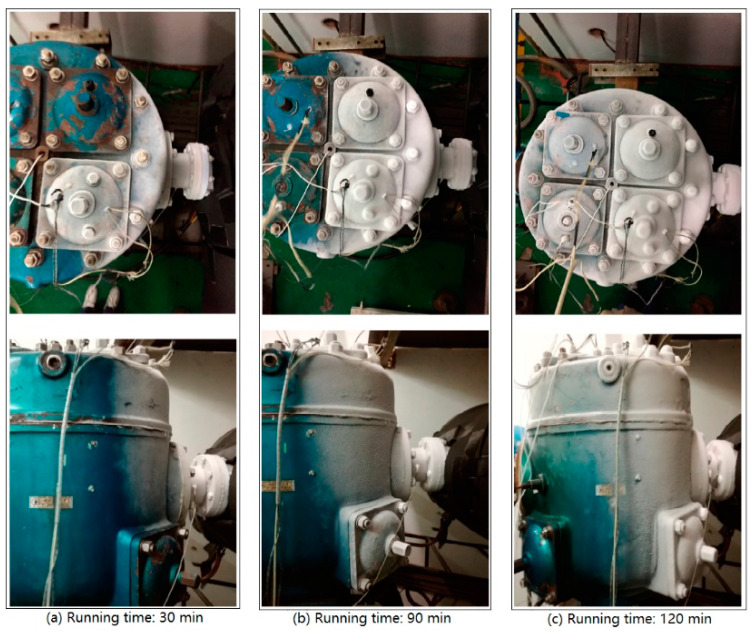
Frosting process of the cylinder.

In this study, only the frost on the outer surface of the cylinder and its influence on the in-compressor thermodynamic process from a macroscopic perspective were observed and analyzed. Figure 16 shows the frost on the top surface of the cylinder head and the side of the cylinder. It can be seen from the figure that the frost first formed near the suction side and that the frost on the head side preceded that on the shaft side. The frost entirely covered the outer surface of the compressor inlet flange at 30 min. The frost had not wholly covered the top of the cylinder head until the run time reached 90 min. The frost did not co-occur on the covers of the two suction valves on the head side, which was the result of the asymmetrical flow field in the cylinder.

### 4.3. Effects of Suction Temperature on a BOG Compressor

Based on the verified FSI model, the effects of suction temperature on a BOG compressor are discussed. Considering that the system used in the paper was started at normal temperature, the upper limit of suction temperature was selected as 30 °C.

#### 4.3.1. Effects of Suction Temperature on Preheating

**Figure 17:**
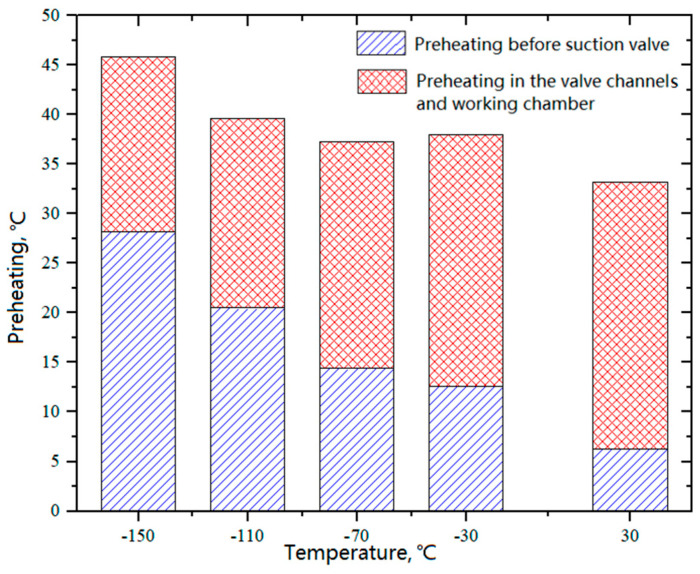
Effects of suction temperature on preheating.

The preheating had two stages at low suction temperatures. The first stage was from the compressor inlet flange to the front of the suction valve. When flowing through the suction chamber, the working fluid was heated by the surrounding air and the inner wall adjacent to the working chamber. In the second stage, when flowing through the valve channels into the working chamber, the working fluid was mixed with the gas in the cylinder and heated by the inner wall of the cylinder and the high-temperature gas leaking from the shift side. The two stages of preheating were simulated at suction temperatures of −150 °C, −110 °C, −70 °C, −30 °C, and 30 °C (see Figure 17). The corresponding preheating before suction valve was simulated as 28.2 °C, 20.5 °C, 14.4 °C, 12.6 °C, and 6.3 °C. The corresponding preheating in the valve channels and working chamber was simulated as 17.6 °C, 19.1 °C, 22.9 °C, 25.4 °C, and 26.9 °C. The variation of preheating with suction temperature can reflect the effects of suction temperature on volumetric efficiency, which directly affects the design of compressor parameters.

#### 4.3.2. Effects of Suction Temperature on Thermodynamic Process

**Figure 18:**
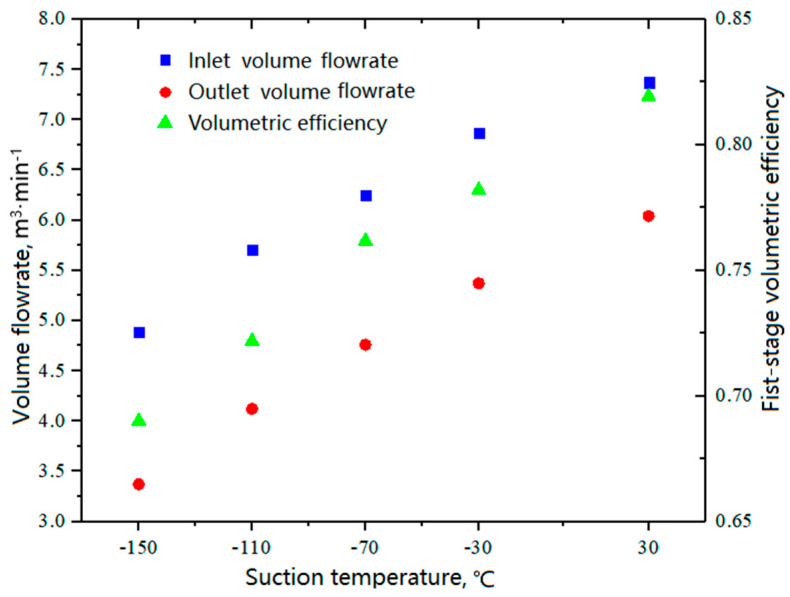
Effects of suction temperature on volume flowrate and volumetric efficiency.

**Figure 19:**
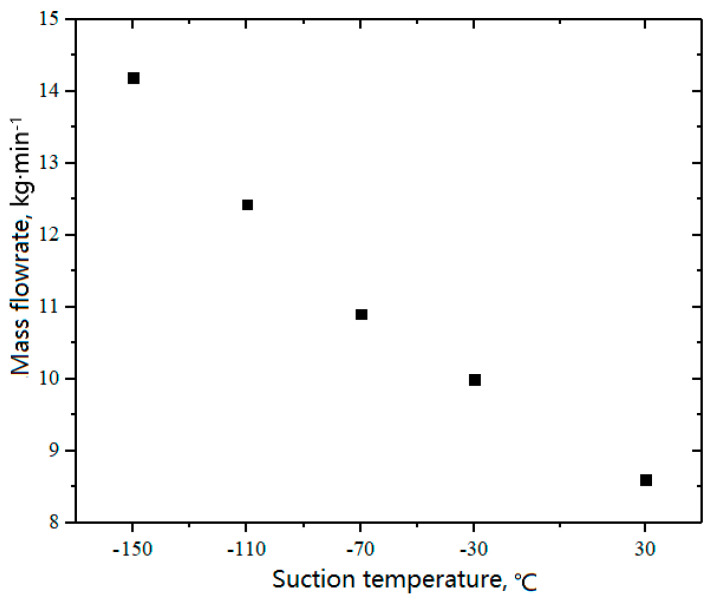
Effects of suction temperature on mass flowrate.

The working fluid used in the experiment was N_2_. The measured inlet and outlet volumetric flowrates of the compressor are shown in Figure 18 under different suction temperatures. Thus, the first-stage volumetric efficiencies were calculated. As the suction temperature decreased from 30 °C to −150 °C, the first-stage volumetric efficiency decreased from 0.82 to 0.69 and the actual outlet flowrate decreased from 6.04 m^3^·min^−1^ to 3.37 m^3^·min^−1^. However, the density of the working fluid increased as the suction temperature decreased, increasing the actual mass flowrate processed by the compressor, as shown in Figure 19. It can also be seen from Figure 17 and Figure 18 that the first-stage volumetric efficiency increased with the decrease in the preheating before suction valve.

#### 4.3.3. Effects of Suction Temperature on Valve Movement

**Figure 20:**
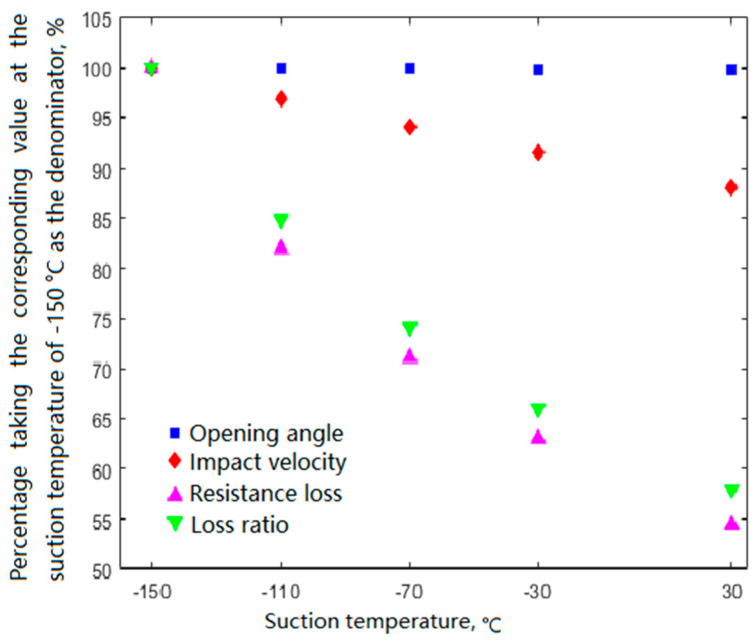
Effects of suction temperature on the operating characteristics of the delivery valve.

Figure 20 presents the simulation results at a suction pressure of 0.03 MPa and a discharge pressure of 0.2 MPa, including the opening angle, impact velocity, resistance loss, and loss ratio of the delivery valve on the head side. The suction temperature had little effect on the opening angle, and the opening was delayed slightly with decreasing suction temperature. As the suction temperature decreased from 30 °C to −150 °C, the impact velocity, resistance loss, and loss ratio increased from 1.614 m·s^−1^ to 1.833 m·s^−1^, from 1.15 kW to 2.11 kW, and from 6.5% to 11.2%, respectively.

## 5. Conclusions

In this study, a numerical model of the internal flow in a BOG compressor and its piping system was established, and a corresponding performance test rig was built. The thermodynamic process inside the working chamber, the pressure pulsation inside the suction/discharge chamber, and the valve motion could be investigated simultaneously by using the proposed FSI model for the reciprocating compressor. The verified 3-D FSI model was used to study the effects of suction temperature on the thermodynamic process and performance of the BOG compressor. As the suction temperature varied, the first-stage volumetric efficiency was inversely related to the preheating before suction valve. Based on the numerical and experimental investigation, the following conclusions can be drawn.

(1) Under low suction temperatures, a frost layer first formed near the suction side, and the frost on the head side preceded that on the shaft side. Moreover, the covers of the two suction valves on the head side did not frost at the same time because of the in-cylinder asymmetrical flow field.
(2) As the suction temperature decreased from 30 °C to −150 °C, the total preheating increased to 45.8 °C, in which the preheating from the compressor inlet flange to the front of the suction valve increased to 28.2 °C, whereas that in the valve channels and working chamber decreased to 17.6 °C.
(3) With the decrease in suction temperature, the volumetric efficiency of the first-stage cylinder decreased from 0.82 to 0.69, whereas the actual mass flowrate processed by the compressor increased because of the increase in the density of the working fluid.
(4) The suction temperature had little effect on the opening angle, whereas the opening angle, impact velocity, resistance loss, and loss ratio all increased when the suction temperature decreased.

Unfortunately, the heat transfer between the cylinder and the internal flow was realized by the given the heat transfer coefficient, and the deformation and the stress distribution could not be evaluated. In the future, we expect to build a fully coupled thermal fluid–structural model, even though the computational cost can be magnificent.

## Figures and Tables

**Table 1:** BOG compressor parameters.

Parameter	Value
Rated rotational speed, rpm	480
Piston stroke, m	0.22
First-stage cylinder diameter, m	0.3
Length of connecting rod, m	0.4
Suction pressure, MPa	0.1
Rate discharge pressure, MPa	0.7
Flowrate, m^3^·min^−1^	11

**Table 2:** Technical parameters of the dynamic pressure sensor.

Parameter	Value
Rated range, MPaA	0–0.35
Operating temperature range, °C	−195.5 to +120
Maximum rated output voltage, mV	100
Rated supply voltage, VDC	10

**Table 3:** Indicated power and power losses in the working chamber of the head side (suction temperature: −150 °C).

	Simulated Value	Experimental Value
Indicated power, kW	18.862	18.143
Suction power losses, kW	2.266	2.133
Discharge power losses, kW	2.110	1.961

## Nomenclature

Symbols	
A	Surface area of a valve plate
$A_{0}$, $A_{s}$	Model Constants for DES
a	Acceleration
$C_{1\varepsilon }$, $C_{2\varepsilon }$	Model Constants for RNG
$C_{1}$, $C_{2}$	Model Constants for DES
$c_{p}$	Specific heat capacity at constant pressure
$\vec{F}$	External body forces
$G_{k}$	Generation of turbulence kinetic energy due to the mean velocity gradients
$G\left(x,x^{\prime }\right)$	Filter function
$\vec{g}$	Gravitational acceleration
H	Valve lift during the movement
$H_{s}$	Precompression length of each spring
I	Unit tensor
K	Spring stiffness
k	Turbulence kinetic energy
$k_{h}$	Thermal conductivity of fluid
m	Mass
N	Number of springs
P	Power
p	Static pressure
$q_{v}$	Volume flowrate of the compressor
$R_{g}$	Gas constant of the working fluid
S	Modulus of the mean rate-of-strain tensor
$S_{T}$	Source term
T	Thermodynamic temperature
t	Time
$\vec{u}$	Fluid velocity
V	Volume of a computational cell; Working volume; Gas volume
$x_{i}$	Cartesian coordinates
$Y_{k}$	Dissipation term
Z	Compressibility coefficient
Greek Symbols	
α	Inverse effective Prandtl numbers
ε	Turbulence dissipation rate; Stage compression ratio
$\eta_{d}$	Electric efficiency
$\eta_{m}$	Mechanical efficiency
$\eta_{t}$	Transmission efficiency
$\eta_{v}$	Volumetric efficiency
$\kappa_{T}$	Adiabatic temperature exponent
$\lambda_{c}$	Purification coefficient
$\lambda_{\varphi }$	Condensation coefficient
μ	Dynamic viscosity
ν	Kinematic viscosity
ρ	Density
σ	Turbulent Prandtl number
$\bar{\phi }\left(x\right)$	Filtered variable
Subscripts	
ad	Adiabatic
d	Discharge
e	Electrical
eff	Effective turbulence
i, j	Components
k	Turbulence kinetic energy
s	Suction
sh	Shift
t	Turbulence
Abbreviations	
BOG	Boil-off gas
CFD	Computational fluid dynamics
DES	Detached eddy simulation
FSI	Fluid–structure interaction
FVM	Finite volume method
LES	Large eddy simulation
LNG	Liquid natural gas
NG	Natural gas
RANS	Reynolds average Navier–Stokes
RNG	Renormalization group
TDC	Top dead center
UDF	User-defined function
